# Relationship between Pachychoroid and Polypoidal Choroidal Vasculopathy

**DOI:** 10.3390/jcm11154614

**Published:** 2022-08-08

**Authors:** Kenji Yamashiro, Yasuo Yanagi, Hideki Koizumi, Hidetaka Matsumoto, Chui Ming Gemmy Cheung, Fumi Gomi, Tomohiro Iida, Akitaka Tsujikawa

**Affiliations:** 1 Department of Ophthalmology and Visual Science, Kochi Medical School, Kochi University, Nankoku 7838505, Japan; 2 Department of Ophthalmology and Visual Sciences, Kyoto University Graduate School of Medicine, Kyoto 6068507, Japan; 3 Department of Ophthalmology and Micro-Technology, Yokohama City University, Yokohama 2320024, Japan; 4 Singapore National Eye Centre, Singapore Eye Research Institute, Singapore 168751, Singapore; 5 The Ophthalmology & Visual Sciences Academic Clinical Program, Duke-NUS Medical School, National University of Singapore, Singapore 169857, Singapore; 6 Department of Ophthalmology, Graduate School of Medicine, University of the Ryukyus, Nishihara 9030215, Japan; 7 Department of Ophthalmology, Gunma University Graduate School of Medicine, Maebashi 3718511, Japan; 8 Department of Ophthalmology, Hyogo College of Medicine, Nishinomiya 6638501, Japan; 9 Department of Ophthalmology, Tokyo Women’s Medical University, Tokyo 1628666, Japan

**Keywords:** age-related macular degeneration, drusen-driven disease, pachychoroid-driven disease, pachychoroid neovasculopathy, polypoidal choroidal vasculopathy

## Abstract

Previous clinical studies have suggested that pachychoroid can induce macular neovascularization (MNV) to develop pachychoroid neovasculopathy (PNV) and that PNV can progress to polypoidal choroidal vasculopathy (PCV). Recent studies based on the pachychoroid concept are now gradually revealing the true nature of, at least some part of, PCV. However, previous studies on PNV and/or PCV have used different frameworks for the classification of PNV, PCV, and neovascular age-related macular degeneration (nAMD). These have hampered the rapid overhaul of the understanding of PCV. Some investigators have assumed that all PCV is pachychoroid-driven whereas other investigators have classified PCV into “pachychoroid PCV” and “non-pachychoroid PCV”. Furthermore, since there is no consensus as to whether PNV includes PCV, some studies have included PCV with PNV, while other studies have excluded PCV from PNV. To address these gaps, we summarize previous studies on PCV and pachychoroid. Even before the proposal of the pachychoroid concept, previous studies had suggested that PCV could be divided into two subtypes, of which one was characterized by pachychoroid features. Previous studies had also provided keys to understand relationship between PCV and PNV. We here recommend a refined conceptual framework for future studies on PNV, PCV, and nAMD. Considering the current inconsistent understanding of PCV, we should be cautious about using the term PCV until we understand the true nature of PCV.

## 1. Introduction

Polypoidal choroidal vasculopathy (PCV) is characterized by polypoidal lesions at the edge of a branching neovascular network and is regarded as a common subtype of neovascular age-related macular degeneration (nAMD) [1]. Other than the unique morphology of macular neovascularization (MNV), PCV has a fairly peculiar pathogenesis. Most importantly, nAMD usually develops on the background of drusen and/or pseudodrusen (subretinal drusenoid deposits, SDD) [2]. In contrast, patients with PCV sometimes lack drusen and SDD, and the mechanisms of developing PCV without drusen/SDD have remained a mystery. However, it seems to be explained, at least in part, by a recently developed concept of a “pachychoroid phenotype”, which defined pachychoroid as a thick choroid. Eyes with a pachychoroid phenotype are frequently associated with dilated outer choroidal vessels (pachyvessels) and/or choroidal vascular hyperpermeability (CVH), and typically lack drusen, except for pachychoroid-associated drusen (pachydrusen). Although recent studies have raised the possibility that the fundamental pathogenesis of pachychoroid disease might involve vortex veins or running patterns of choroidal vessels, the term “pachychoroid” is widely used and the definition of pachychoroid has not been established.

**Figure 1:**
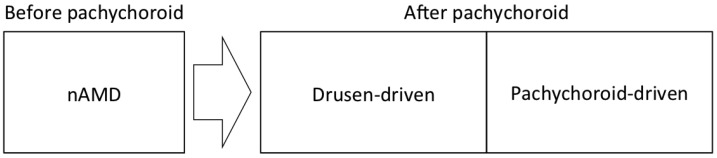
The framework for the subtypes of neovascular age-related macular degeneration (nAMD) in the era of the pachychoroid concept. Exudative maculopathy, previously diagnosed as nAMD, is divided into drusen-driven disease and pachychoroid-driven disease after the development of the pachychoroid concept.

The concept of pachychoroid classified nAMD into subtypes of pachychoroid-driven disease and drusen-driven disease (Figure 1), as we detailed previously [3,4]. However, the diagnostic criteria for pachychoroid- (and drusen-) driven diseases have not been fully established to date, and the border between these two disease spectrums remains ambiguous. There is an urgent need to refine the diagnostic criteria for these two subtypes. Additionally, further studies need to scrutinize cases that seem to belong to both spectrums, and/or cases that seem not to belong to either spectrum. Future studies might establish a third subgroup that is neither pachychoroid-driven nor drusen-driven. For future studies to refine this classification, investigators have to use the same framework.

From the perspective of pachychoroid, the classification of PCV also needs to be revisited. nAMD is often classified into PCV, retinal angiomatous proliferation (RAP), and typical nAMD, mainly based on morpho-angiological studies [5,6]. Since RAP usually accompanies drusen/SDD and a thin choroid, most RAP should be classified as drusen-driven and not pachychoroid-related. Typical nAMD would be classified into either drusen- or pachychoroid-driven. However, opposing perspectives seem to exist for PCV. Some investigators assume that almost all PCV is pachychoroid-driven, whereas other investigators classify PCV into “pachychoroid PCV” and “non-pachychoroid PCV”. As detailed in this review, previous studies had suggested the existence of both “pachychoroid PCV” and “non-pachychoroid PCV” even before the proposal of the pachychoroid concept. Previous reports on the link between PCV and central serous chorioretinopathy (CSC), a common subtype of pachychoroid disease, had suggested that a small proportion of PCV developed on abnormalities secondary to CSC or subclinical CSC. Furthermore, previous studies had proposed various dichotomous classification schemes of PCV, suggesting that PCV could be classified into “pachychoroid PCV” and “non-pachychoroid PCV”.

In addition, there is another gap in the literature regarding the classification and nomenclature of PCV, which is causing more confusion. There is no consensus as to whether PCV is a distinct entity in the pachychoroid-driven MNV or should be regarded as a variant of pachychoroid-driven MNV. For example, to evaluate treatment outcomes of pachychoroid-driven MNV, some studies included PCV while other studies excluded PCV. The inconsistent classification of PCV in previous studies has hampered the rapid overhaul of the understanding of PCV in the context of pachychoroid. To address these gaps, we here present an updated compendium of previous studies on PCV and pachychoroid to understand their relationships and recommend a refined framework for future studies on these diseases. It is hoped that investigators use the same framework to classify PCV and its associated diseases.

## 2. Vascular Lesion of PCV

It has now been established that the branching neovascular network (formally designated as branching vascular network) in PCV represents type 1 MNV. It had been long debated whether the vascular lesion of PCV was choroidal neovascularization (CNV: now referred to as MNV [2]) or choroidal vascular abnormality. The term “polypoidal CNV” was proposed in 1999 [7], and the term “polypoidal CNV” has been used to describe the vascular lesion of PCV or as a substitute for the disease name of PCV [8,9,10,11,12]. Furthermore, the term “branching neovascular network” was recently recommended to replace the previous term “branching vascular network”, considering that PCV is a variant of type 1 neovascularization within the nAMD spectrum [1]. PCV is also called aneurysmal type 1 MNV.

## 3. Subtype Classification of PCV

To capture the true nature of the underlying pathogenesis of PCV, many study groups have divided PCV into two subtypes and evaluated the differences between the two subtypes. Although most classification was proposed before the proposal of the pachychoroid concept, previous dichotomous classifications seemed to be based on what is now known as pachychoroid characteristics.

### 3.1. Type 1 and Type 2 PCV

In 2005, Yuzawa et al. reported that most cases with PCV could be classified into two subtypes, “PCV in the narrow sense” and “POLYPOIDAL CNV” (Table 1) [13,14]. Since “polypoidal CNV” had been originally proposed for CNV in PCV or PCV itself and Yuzawa et al. used “POLYPOIDAL CNV” differently, “POLYPOIDAL CNV” by Yuzawa et al. is written in uppercase for differentiation. They defined that PCV in the narrow sense is the typical and true PCV, consisting of inner choroidal vessel abnormalities, not CNV, whereas POLYPOIDAL CNV expands under the retinal pigment epithelium (RPE), ultimately with polypoidal lesions developing at vessel termini. Later, in 2011, they demonstrated that the genetic background of “POLYPOIDAL CNV” was similar to that of nAMD, while the genetic background of “PCV in the narrow sense” was different from that of nAMD and was rather similar to that of normal controls [15]. It was suggested that “POLYPOIDAL CNV” belongs to nAMD but that “PCV in the narrow sense” has a different pathogenesis. In 2013, they proposed the terms type 1 PCV for “POLYPOIDAL CNV” and type 2 PCV for “PCV in the narrow sense [16]”. They presumed a contribution of CVH to the development of type 2 PCV [16] and demonstrated a thicker choroid in type 2 PCV [17]. Although they proposed this classification before the proposal of the pachychoroid concept, type 2 PCV seems to belong to pachychoroid disease.

Although later studies did not support the vascular abnormality as the origin of type 2 PCV, the terms of type 1 PCV and type 2 PCV have been instilled and have penetrated, particularly in Asia where most patients with nAMD undergo indocyanine green angiography (ICGA), and many studies have evaluated the difference between type 1 PCV and type 2 PCV (Table 2). Type 1 PCV was reportedly observed in 26–60% (median 45%) and type 2 PCV in 40–74% (median 55%) of eyes with PCV [17,18,19,20,21,22,23,24,25]. The genetic background of type 1 PCV was similar to the genetic background of nAMD, but the genetic background of type 2 PCV was different from the genetic background of nAMD [18]. Three studies from Japan reported larger lesion sizes in type 1 PCV than in type 2 PCV [17,20,23]. The incidence of fibrotic scars after anti-VEGF treatment was higher in type 1 PCV than in type 2 PCV [25]. Unfortunately, this old classification did not seem to gain popularity outside Asia; however, it provided strong evidence that there might be two distinct subtypes within PCV and provided important momentum to investigate the underlying differences between these two subtypes.

### 3.2. Other Classification

Other classifications of PCV are similar to those proposed by Yuzawa et al. but are simpler than the original classification. These classification systems are also based on the idea that PCV is a group of heterogenous diseases consisting of those with “nAMD”-like features and those without. In 2011, Tsujikawa et al. evaluated the difference between “larger” PCV and “smaller” PCV (Table 3) [26]. PCV with vascular lesion sizes smaller than one disc area showed minimal progression of the lesion, with better visual prognosis, whereas PCV with vascular lesion sizes larger than one disc area showed significant progression of the lesion, with worse visual prognosis. Subfoveal fibrosis was seen more frequently in larger PCV. Furthermore, the genetic background of larger PCV was similar to that of nAMD, while the genetic background of smaller PCV was more similar to that of normal subjects. The lesion size and the genetic background suggest that larger PCV is similar to type 1 PCV and that smaller PCV is similar to type 2 PCV. In 2015, Coscas et al. classified PCV into “nAMD–related polyps” and “idiopathic polyps” referring to patient age, drusen, hemorrhagic pigment epithelial detachment (PED), fluorescein angiography (FA) leakage, branching vascular network on ICGA, optical coherence tomography (OCT) findings, lesion location, lesion number, and evolution rapidity (Table 4) [27]. Eyes with “nAMD-related polyps” had a larger lesion size, more drusen, and thinner choroid than eyes with “idiopathic polyps”. It seems that “nAMD-related polyps” have characteristics of drusen-driven nAMD while “idiopathic polyps” have characteristics of pachychoroid. Essentially, these classifications further reinforced the hypothesis that PCV can be categorized into two subgroups.

## 4. Relationship between PCV and CSC

Even before the development of the pachychoroid concept, it had been suggested that pathogenesis of CSC might contribute to the development of some parts of PCV from various viewpoints. Phenotypic similarity between PCV and CSC had suggested a possible common pathogenesis of these two conditions. The history of CSC among patients with PCV had also suggested a possible common pathogenesis of PCV and CSC, although few patients with PCV had a history of CSC. Furthermore, studies on CVH had suggested the contribution of “subclinical” CSC to the development of PCV, although few patients with PCV had CVH. These studies supported the hypothesis that PCV develops in eyes with “CSC-like” features, but it is worth mentioning that only a small portion of PCV (35% at most) had distinct CSC features, as detailed below.

### 4.1. Phenotypic Similarity between PCV and CSC

Since chronic CSC can develop into type 1 MNV with serous retinal detachment, subretinal hemorrhage, and small PEDs, fundus examination and FA are sometimes insufficient to differentiate CSC from PCV with serous retinal detachment, subretinal hemorrhage, and polypoidal lesions. Yannuzzi et al., in 2000, recommended ICGA examination to detect polypoidal lesions for differentiation of PCV from CSC [9]. However, polypoidal lesions of PCV spontaneously resolve or regress with treatment. As several studies have pointed out, it is difficult to differentiate CSC from PCV without polypoidal lesions, even with ICGA [11,28,29]. In 2009, Hikichi et al. raised the possibility that we could encounter early-stage PCV without polypoidal lesions that have evolved from CSC [28]. This concept of early-stage PCV would suggest pachychoroid-driven MNV as a mechanism for developing PCV. In 2010, Ooto et al. suggested that the pathogenesis of PCV and CSC was similar in part, in their study comparing phenotypes of PCV and CSC [29].

### 4.2. History of CSC in Patients with PCV

In 1999, a study group reported a case series of chronic CSC patients, who later developed PCV [30], and in 2001 they suggested the possibility that long-standing changes in the RPE in chronic CSC predisposed eyes to the formation of polypoidal lesions in PCV [31]. In 1992, a study by the Japanese Ministry of Health and Welfare reported a frequent CSC history in patients with nAMD [32]. Half of these investigated Japanese patients with nAMD could have developed PCV, given that later studies revealed that the prevalence of PCV in Japanese nAMD was about 50% [5,6]. In 2009, a more frequent CSC history was reported in Japanese patients with PCV (15%) than in patients with typical nAMD (3%) [33].

### 4.3. CVH in PCV

CVH and thicker choroid have been regarded as hallmarks of CSC. In contrast to CSC, in 2000 Yannuzzi et al. reported that none of the 13 patients with PCV presented CVH in late ICGA [9]. However, a later study from Japan found that 10% of eyes with PCV exhibited CVH and presumed that abnormalities in eyes with chronic CSC or subclinical CSC might contribute to the occurrence of PCV in 2006 [34]. In 2013, Koizumi et al. reported a frequency of CVH of 35% in Japanese patients with PCV and suggested a shared pathogenic mechanism between CSC and PCV [35]. They also reported that 26% of PCV cases with and 5% of PCV cases without CVH had a CSC history.

CVH can also be observed in typical nAMD patients, and its associations with treatment outcomes for typical nAMD and/or PCV have been evaluated in several studies (Table 5). Some studies have reported favorable treatment outcomes for eyes with CVH, whereas other studies have reported favorable treatment outcomes for eyes without CVH. There is no clear association of CVH with the treatment outcome for typical nAMD and/or PCV.

Together, these studies investigating the link between CSC and PCV demonstrated that PCV may develop in eyes with a CSC background, i.e., PCV may occur not only secondary to CSC but also secondary to “CSC-like changes or forme fruste CSC”. This led to the concept of pachychoroid being a precursor of PCV.

## 5. Establishment of the Pachychoroid Concept

The concept of pachychoroid, particularly the hypothesis that defines pachychoroid as a precursor of type 1 MNV with or without polypoidal lesions, has revolutionized our understanding of PCV pathology. At the same time, however, it has created some confusion among ophthalmologists about PCV. Firstly, about the pathogenesis. Are all PCVs pachychoroid-driven or are only some parts of PCV that are pachychoroid-driven? Secondly, about the nomenclature. Is pachychoroid-driven PCV a distinct disease entity or simply an extension of pachychoroid-driven MNV, implying that these should be grouped together as one category?

### 5.1. Proposal of the Pachychoroid Concept

In 2012, Fung et al. reported a case series of type 1 MNV associated with choroidal thickening that could be misinterpreted as nAMD [46]. In 2013 Warrow et al. introduced the term pachychoroid neovasculopathy (PNV) for this entity [47]. Exudative maculopathy, previously diagnosed as nAMD, is now divided into drusen-driven nAMD and PNV (Figure 1). Together with the introduction of PNV, Warrow et al. presented nine cases of pachychoroid pigment epitheliopathy (PPE). PPE is an incomplete form of CSC, without serous macular detachment and a history of subretinal fluid. They suggested that PPE fell within the pathophysiologic spectrum of CSC. Eyes with PPE presented increased choroidal thickness, reduced fundus tessellation, and RPE abnormalities with or without small PEDs overlying localized areas of thickened choroid and/or dilated outer choroidal vessels, i.e., pachyvessels. In 2015, Pang et al. reported three cases of PNV and described the detailed characteristics of PNV [48]. They suggested that PNV fell within a spectrum of diseases associated with choroidal thickening that includes PPE, CSC, and PCV. The type 1 MNV of PNV overlay a localized area of choroidal thickening and/or pachyvessels, and CVH was observed beneath the area of the CNV. Eyes with PNV lacked drusen, except for pachydrusen [49]. Although polypoidal lesions were observed in all three cases of PNV reported by Pang et al. and none of these cases had a history of CSC, later studies included eyes without polypoidal lesions and eyes with a history of CSC in PNV [50,51,52].

### 5.2. Evolution of the Pachychoroid Concept

The concept of pachychoroid diseases was developed subjectively, depending on the observation of diseases by experienced clinicians, and its purported role in the pathogenesis of exudative maculopathies was being questioned. However, later studies objectively confirmed the genetic differences between pachychoroid diseases and nAMD and demonstrated the clinical significance of the pachychoroid concept using artificial intelligence (AI). This supported that there are at least two subgroups of exudative maculopathy, of which one is characterized by pachychoroid features.

In 2018, a genome-wide association study discovered an association between *CFH* and pachychoroids in Japanese subjects [53], and a later study from South Korea confirmed this association [54]. It was well known that the G allele of *CFH* I62V was a risk allele for drusen-driven nAMD, while the A allele of *CFH* I62V was a protective allele for this condition. In contrast, the G allele of *CFH* I62V was identified as a protective allele for pachychoroid, while the A allele of *CFH* I62V was found to be a risk allele for pachychoroid. These findings indicated that pachychoroid-related diseases are genetically different from drusen-related diseases. In 2020, a study from Japan further confirmed the significance of the pachychoroid concept by using AI [55]. Analysis of 537 nAMD cases with deep phenotype unsupervised machine learning demonstrated that nAMD may be divided into two clusters, focusing on the features that experienced clinicians had subjectively proposed as characteristics of pachychoroid. AI also supported the differentiation of PNV from drusen-driven nAMD. In addition to the confirmation of the significance of the pachychoroid concept, this machine learning analysis revealed key features for dividing nAMD into PNV and drusen-driven nAMD. Although a concrete definition of pachychoroid has not yet been established, the machine learning-assisted definition of pachychoroid is summarized in Table 6. It should be noted that this machine learning analysis did not choose the existence of polypoidal lesions as an important factor for the clustering of nAMD.

### 5.3. Polypoidal Lesions in PNV

Some investigators have recognized that PNV and a branching neovascular network in PCV are synonymous. Additionally, polypoidal lesions seem to represent an expansion of PNV. Since there is no consensus as to whether the terminology “PNV” includes eyes with polypoidal lesions, initially most investigators considered that PNV includes both pachychoroid-driven MNV with polypoidal lesions and those without. However, recent studies often regarded only pachychoroid-driven MNV without polypoidal lesions as PNV. Such investigators called pachychoroid-driven MNV with polypoidal lesions PCV.

Most studies on PNV during the period 2012–2018 included eyes with polypoidal lesions in PNV. The original case series of PNV from the US reported that all three eyes showed the appearance of polypoidal structures within the neovascular tissue [48]. Other studies from the US used the terms “nonpolypoidal PNV” or “PNV without polypoidal features” for PNV without polypoidal lesions and “polypoidal PNV” or “PNV with PCV” for PNV with polypoidal lesions [56,57]. The prevalence of polypoidal lesions in PNV was reportedly 17–36% in the US (Table 7). The first study on PNV from Japan reported that the prevalence of polypoidal lesions was 56% in PNV and 43% in nAMD (*p* = 0.11), revealing that at least 20% of eyes previously diagnosed as nAMD were PNV [50]. Other later studies from Japan reported that the prevalence of polypoidal lesions was 44% in PNV and 33% in nAMD (*p* = 0.69) [51], 67% in PNV and 50% in nAMD with type 1 CNV (*p* = 0.09) [52], and 22% in PNV and 50% in nAMD with type 1 CNV (*p* = 0.16) [58]. A case series of PNV from Europe included two eyes with polypoidal lesions (40%) among their five cases of PNV [59].

Although some recent studies continued to include eyes with polypoidal lesions under PNV, other recent studies from Japan, South Korea, and Europe excluded eyes with polypoidal lesions from PNV. Even after 2019, several studies from Japan have included eyes with polypoidal lesions in PNV and have compared PNV with polypoidal lesions and those without polypoidal lesions. The reported prevalence of polypoidal lesions was 48–56% (Table 7) [60,61,62,63]. However, several other studies from Japan have performed their analysis, limiting their study target only to PNV without polypoidal lesions [64,65,66,67,68,69,70,71]. Among these studies, one study considered that PNV included both PNV with and those without polypoidal lesions, and they performed their analysis only on PNV without polypoidal lesions [64]. Other studies considered PNV without polypoidal lesions as PNV, and PNV with polypoidal lesions as PCV [67,68,69,70,71]. In 2019, a study from South Korea compared anti-VEGF treatment outcomes for PNV and nAMD after excluding eyes with polypoidal lesions [72]. Although this study did not deny the inclusion of eyes with polypoidal lesions in PNV, another study on PNV from South Korea excluded eyes with polypoidal lesions from PNV, based on their understanding that type 1 CNV with polypoidal lesions should be classified as PCV [73]. Later studies from Korea have also considered PNV without polypoidal lesions as PNV and PNV with polypoidal lesions as PCV [74,75,76,77]. In Europe, an Italian study group included eyes with polypoidal lesions in PNV [78], whereas study groups from Germany and Turkey excluded eyes with polypoidal lesions from PNV [79,80,81,82,83].

## 6. Treatment Response of PNV

Although many previous studies have compared treatment outcomes between nAMD and PNV, clear differences have not been confirmed. It seems that the current immature definition of PNV and PCV has hampered the comparison of treatment response between nAMD and PNV. It is impossible for all retina specialists’ subjective judgments to agree perfectly on whether an eye has pachychoroid characteristics because objective criteria have not been established for diagnosing pachychoroid, pachyvessels, or pachydrusen. Considering that previous studies on pachychoroid diseases used slightly different criteria for their diagnosis and as their diagnosis depended on subjective judgment, previous studies on pachychoroid diseases have not evaluated exactly the same disease. Furthermore, some previous studies included pachychoroid-driven MNV with polypoidal lesions in PNV, while other studies excluded such cases from PNV.

Several previous studies compared treatment responses between PNV and nAMD by including eyes with polypoidal lesions in these two groups (Table 8). There seems to be no clear difference in fluid control and visual acuity (VA) changes after anti-VEGF treatment for PNV and nAMD [50,52,58,60,62]. Other studies evaluated treatment responses of PNV with or without polypoidal lesions and reported that photodynamic therapy (PDT) and anti-VEGF treatment were effective for PNV [63,84]. PNV with polypoidal lesions might require less aflibercept treatment [52], while PNV without polypoidal lesions might require less PDT [63]. To date, only one group from South Korea compared PNV and nAMD by excluding eyes with polypoidal lesions from the studied cohorts (Table 9). This group suggested no clear difference in fluid control and visual acuity change after anti-VEGF treatment for PNV and nAMD [72,77]. Other studies on PNV without polypoidal lesions have reported that PDT and anti-VEGF treatment were effective for PNV without polypoidal lesions [64,65,66,73,80,81,85,86]. In 2019 and 2021, studies from Japan compared the 5-year treatment outcomes after PDT or anti-VEGF treatment for PCV with and without pachychoroid (Table 10) [87,88]. The baseline VA was not significantly different between the two groups, and the final VA was reportedly better in cases of PCV with pachychoroid after PDT, but the final VA was not significantly different between the two groups after anti-VEGF treatment.

## 7. Relationship between PNV and PCV

**Figure 2:**
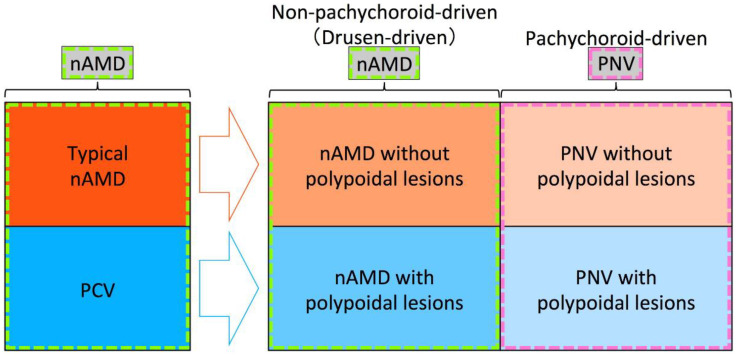
Recommended framework for future studies on pachychoroid diseases. Among exudative maculopathy previously diagnosed as neovascular age-related macular degeneration (nAMD), typical nAMD is divided into nAMD without polypoidal lesions and pachychoroid neovasculopathy (PNV) without polypoidal lesions, and polypoidal choroidal vasculopathy (PCV) is divided into nAMD with polypoidal lesions and PNV with polypoidal lesions, after the development of the pachychoroid concept. In the era of the pachychoroid concept, nAMD includes non-pachychoroid-driven (drusen-driven) nAMD with and without polypoidal lesions, and PNV includes PNV with and without polypoidal lesions.

**Figure 3:**
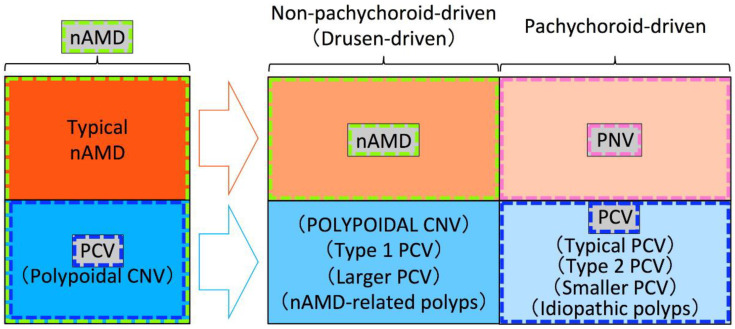
An example of the framework used in previous studies. Among exudative maculopathy previously diagnosed as nAMD, typical nAMD is divided into nAMD that is non-pachychoroid-driven (drusen-driven) and pachychoroid neovasculopathy (PNV) that is pachychoroid-driven, while polypoidal choroidal vasculopathy (PCV) is divided into macular neovascularization (MNV) with polypoidal lesions that is non-pachychoroid-driven (drusen-driven) and PCV that is pachychoroid-driven, in the era of the pachychoroid concept. nAMD includes only non-pachychoroid-driven MNV without polypoidal lesions, PNV includes only pachychoroid-driven MNV without polypoidal lesions, and PCV includes only pachychoroid-driven MNV with polypoidal lesions. An appropriate term for non-pachychoroid-driven MNV with polypoidal lesions has not yet been proposed. Since the “polypoidal CNV” had been proposed for CNV in PCV or PCV itself and the usage of “POLYPOIDAL CNV” by Yuzawa et al. differs from the originally used term “polypoidal CNV”, “POLYPOIDAL CNV” by Yuzawa et al. is written in uppercase for differentiation.

**Figure 4:**
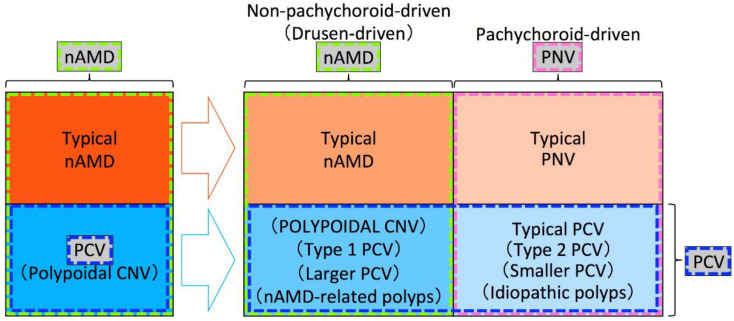
An example of a framework used in previous studies. Among exudative maculopathy previously diagnosed as nAMD, typical nAMD is divided into typical nAMD that is non-pachychoroid-driven (drusen-driven) and typical pachychoroid neovasculopathy (PNV) that is pachychoroid-driven, and polypoidal choroidal vasculopathy (PCV) is divided into non-pachychoroid-driven (drusen-driven) macular neovascularization (MNV) with polypoidal lesions and pachychoroid-driven MNV with polypoidal lesions, in the era of the pachychoroid concept. nAMD includes both non-pachychoroid-driven MNV without and with polypoidal lesions. PNV includes both pachychoroid-driven MNV without and with polypoidal lesions. PCV includes both non-pachychoroid-driven MNV with polypoidal lesions and pachychoroid-driven MNV with polypoidal lesions. Pachychoroid-driven MNV with polypoidal lesions should be called typical PCV and an appropriate term for non-pachychoroid-driven MNV with polypoidal lesions has not yet been proposed. Since the “polypoidal CNV” had been proposed for CNV in PCV or PCV itself and the usage of “POLYPOIDAL CNV” by Yuzawa et al. differs from the originally used term “polypoidal CNV”, “POLYPOIDAL CNV” by Yuzawa et al. is written in uppercase for differentiation.

In this section, we will describe important insights yielded by pachychoroid research, and some strategies which may help improve our understanding of PCV in the context of pachychoroid. The fact that previous studies have not used the same framework for the classification of MNV is problematic, and the resultant ambiguity has been undermining the clinical usefulness of the conceptual classification using pachychoroid features. We insist that these problems can be addressed only by performing future studies standing on the same framework. Although the delineation between nAMD and PNV remains ambiguous due to the currently immature diagnostic criteria, understanding the true nature of nAMD, PNV, and PCV in the future will refine this distinction. As such, for future studies, we would like to recommend the framework shown in Figure 2. Since previous studies used various frameworks that were similar to those in Figure 3 or Figure 4, we will first point out ambiguities of frameworks in Figure 3 and Figure 4, and then explain the reasonableness of the framework in Figure 2. Given that most RAP belongs to drusen-driven MNV, RAP is excluded from these figures.

### 7.1. Does PCV Continue to Include Both Pachychoroid PCV and Non-Pachychoroid PCV?

The original report introducing the pachychoroid concept speculated that PPE, CSC, and some eyes with PCV fell within a spectrum of pachychoroid-related disease [47]. It is suggested that some eyes with PCV belong to pachychoroid disease, while other eyes with PCV belong to non-pachychoroid disease. At least some part of the non-pachychoroid PCV would develop on drusen-driven mechanisms. Previously proposed “typical PCV”, “type 2 PCV”, “smaller PCV,” and “idiopathic polyps” would fall into pachychoroid-driven PCV, while “POLYPOIDAL CNV”, “type 1 PCV”, “larger PCV,” and “nAMD-related polyps” would fall into non-pachychoroid-driven (drusen-driven) PCV.

Although previous studies on the dichotomous classification of PCV seem to suggest that pachychoroid-driven PCV possesses the true nature of PCV, it has not been determined whether both pachychoroid-driven PCV and non-pachychoroid-driven PCV should be called PCV or only pachychoroid-driven PCV should be called PCV in the era of the pachychoroid concept. We cannot determine which is appropriate until we understand the true nature of PCV. If we restrict the usage of the term PCV to pachychoroid-driven PCV as in Figure 3, we would have to identify a new term for non-pachychoroid-driven PCV. If we use the term PCV for both non-pachychoroid-driven PCV and pachychoroid-driven PCV as in Figure 4, it would be practical to use “typical PCV” for pachychoroid-driven PCV and a new term for non-pachychoroid-driven PCV.

As for the new term for non-pachychoroid-driven PCV, it should not be “polypoidal CNV” because “polypoidal CNV” had been originally proposed for PCV that had included both non-pachychoroid-driven PCV and pachychoroid-driven PCV. Furthermore, type 1 CNV with polypoidal lesions can be observed in various diseases regardless of ethnicity, such as angioid streaks, dome-shaped macula, inferior staphyloma, and choroidal nevus [89,90,91,92,93]. Balaratnasingam et al. proposed to use “polypoidal CNV” for all these type 1 CNV with polypoidal lesions except for pachychoroid-driven PCV [94]. Since “polypoidal CNV” has been used for and proposed for various disease conditions, it would be better to avoid using “polypoidal CNV” in future studies.

### 7.2. Does nAMD Include Both Eyes without Polypoidal Lesions and Eyes with Polypoidal Lesions in the Era of the Pachychoroid Concept?

**Figure 5:**
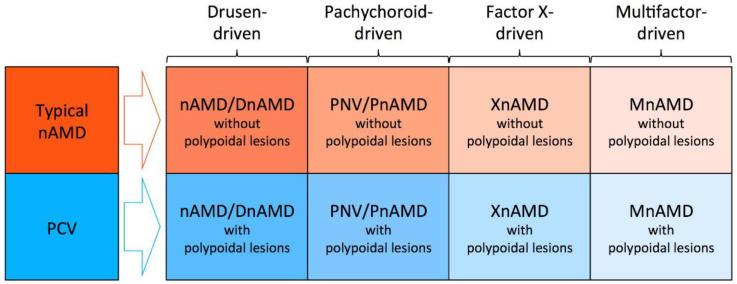
Possible future framework for neovascular age-related macular degeneration (nAMD). Typical nAMD might be divided into drusen-driven nAMD without polypoidal lesions, pachychoroid neovasculopathy (PNV) without polypoidal lesions, factor X-driven AMD without polypoidal lesions, and multifactor-driven mAMD without polypoidal lesions.

In the era of the pachychoroid concept, typical nAMD is also divided into two subtypes of non-pachychoroid-driven disease and pachychoroid-driven disease. Considering that most non-pachychoroid-driven typical nAMD are likely to develop on drusen-driven mechanisms, “drusen-driven” would be a more appropriate term for non-pachychoroid-driven typical nAMD. However, this subgroup might be re-named or further classified into multiple subgroups if future studies reveal that drusen are not the essential mechanisms for this subgroup or find new mechanisms other than drusen-driven mechanisms (Figure 5).

As for the usage of the term nAMD, nAMD had included eyes with polypoidal lesions (PCV) and eyes without polypoidal lesions (typical nAMD) before the proposal of the pachychoroid concept. However, in the era of the pachychoroid concept, it has not been determined whether nAMD includes both eyes with polypoidal lesions and eyes without polypoidal lesions or nAMD includes only eyes without polypoidal lesions. If we include both subtypes into nAMD as in Figure 4, drusen-driven nAMD without polypoidal lesions should be called “typical nAMD” to be differentiated from drusen-driven nAMD with polypoidal lesions. Instead, we might be able to restrict the usage of nAMD only for drusen-driven nAMD without polypoidal lesions (Figure 3).

### 7.3. Does PNV Include Both Eyes without Polypoidal Lesions and Eyes with Polypoidal Lesions?

Similar to the usage of the term nAMD, it has not been determined whether PNV includes both eyes with polypoidal lesions and eyes without polypoidal lesions or PNV includes only eyes without polypoidal lesions. The original case report of PNV described that all three eyes with PNV showed the appearance of polypoidal structures within the neovascular tissue, suggesting that PNV includes PNV with polypoidal lesions (Figure 4) [48]. However, the same original case report concluded that PNV could ultimately progress to the development of PCV, suggesting that pachychoroid-driven MNV without polypoidal lesions should be called PNV, while pachychoroid-driven MNV with polypoidal lesions should be called PCV. If we include both eyes with polypoidal lesions and eyes without PNV as in Figure 4, pachychoroid-driven MNV without polypoidal lesions should be called “typical PNV” to be differentiated from pachychoroid-driven MNV with polypoidal lesions. Instead, we might be able to restrict the usage of PNV only for pachychoroid-driven MNV without polypoidal lesions (Figure 3).

### 7.4. Proposed Nomenclature for nAMD, PCV, and PNV

Until we understand the true nature of PCV, we should be cautious about using the term PCV. Before the proposal of the pachychoroid concept, many clinical and genetic studies evaluated the differences between typical nAMD and PCV. However, it has not been clearly demonstrated whether the previously reported differences are the differences between non-pachychoroid-driven MNV and pachychoroid-driven MNV or the differences between MNV without polypoidal lesions and MNV with polypoidal lesions. If there are clear clinical or genetic differences between PNV with polypoidal lesions and PNV without polypoidal lesions, it could be rational to use “PNV” only for PNV without polypoidal lesions and call PNV with polypoidal lesions “PCV”. However, previous studies have not yet demonstrated clear differences. At least until such differences are identified, it would be adequate to use the terms “PNV with polypoidal lesions” and “PNV without polypoidal lesions” for these two categories, as shown in Figure 2. Additionally, as for drusen-driven nAMD, it could be rational to use “nAMD” only for nAMD without polypoidal lesions, and “PCV” or another new term for nAMD with polypoidal lesions, if there is a clear difference between nAMD with and without polypoidal lesions. However, previous studies have not clearly demonstrated such differences. For near-future studies, it would be adequate to use the terms “nAMD with polypoidal lesions” and “nAMD without polypoidal lesions” for these two categories, as shown in Figure 2.

Polypoidal lesions of PCV spontaneously resolve or regress with treatment, while nAMD without polypoidal lesions can develop polypoidal lesions [95,96]. This appearance and disappearance of polypoidal lesions would also support the framework in Figure 2. If we use different terms for eyes with and eyes without polypoidal lesions, we have to change the diagnosis every time an eye develops polypoidal lesions, as well as every time the polypoidal lesions regress. Furthermore, it would be difficult to judge whether eyes have polypoidal lesions or not during the transitional period from eyes with no polypoidal lesions to eyes with obvious polypoidal lesions.

Two clustering studies of nAMD also suggested that the existence of polypoidal lesions was not an important factor for clustering. A study using deep phenotype unsupervised machine learning revealed that nAMD diagnosed before the pachychoroid concept should be divided into two clusters of drusen-driven MNV and pachychoroid-driven MNV, not into four clusters, and the two clusters showed a similar prevalence of polypoidal lesions, suggesting that polypoidal lesions are not an important factor in clustering of nAMD and PNV [55]. A study from Korea performed a clustering analysis of choroidal profiles and found that PNV with and PNV without polypoidal lesions were grouped in the same cluster [97].

## 8. Future Directions

To establish a complete pachychoroid concept, future studies standing on the framework as shown in Figure 2 should elucidate whether the previously reported differences between typical nAMD and PCV are differences between drusen-driven disease and pachychoroid-driven disease, or differences between eyes with and eyes without polypoidal lesions. When the term PCV is used in these future studies, it is necessary to describe whether it includes both nAMD with polypoidal lesions and PNV with polypoidal lesions or it includes only PNV with polypoidal lesions. Once the true nature of nAMD and PNV, and the significance of polypoidal lesions have been elucidated, it would be possible to complete a reasonable framework for nAMD, PNV, and PCV.

Although the pachychoroid concept was developed with consideration of a thickened choroid, pachyvessels, and CVH, recent studies have raised the possibility that asymmetric choroidal vessels, asymmetric vortex veins, dilated vortex veins, and/or vortex vein congestion are involved in the pathogenesis of pachychoroid disease [68,69,70,71]. The fundamental pathogenesis of pachychoroid disease might involve vortex veins or running patterns of choroidal vessels. Since future studies might discover the true nature of pachychoroid diseases and may change the diagnostic criteria for pachychoroid diseases, it is necessary to continue studies using a pliable framework for the subtype classification of nAMD and PNV, as shown in Figure 2.

## Figures and Tables

**Table 1:** Definition and characteristics of proposed typical PCV and POLYPOIDAL CNV.

	POLYPOIDAL CNV	Typical PCV
Yuzawa et al. [13]	Polypoidal lesions developing at termini of CNV under the RPE	Inner choroidal vessel abnormality
Yuzawa et al. [14]	*N* = 20 (51%)	*N* = 19 (49%)
	Choroidal thickness = 212 ± 84 µm	Choroidal thickness = 348 ± 91 µm
	Both feeder and draining vessels are visible, and network vessels show characteristic findings of CNV in ICGA.	Neither feeder nor draining vessels are visible, and there are few network vessels in ICGA.
	GLD in ICGA = 4759 ± 1655 µm	GLD in ICGA = 1798 ± 738 µm
		PCV in the narrow sense
Tanaka et al. [15]	*N* = 85 (30%)	*N* = 202 (70%)
	Significant associations with *CFH* I62V and *ARMS2* A69S	Significant association with *CFH* I62V but not with *ARMS2* A69S
Kawamura et al. [16]	*N* = 13 (42%)	*N* = 18 (58%)
	Choroidal thickness = 199 ± 65 µm	Choroidal thickness = 288 ± 98 µm
	Type 1 PCV	Type 2 PCV

PCV: polypoidal choroidal vasculopathy, CNV: choroidal neovascularization, RPE: retinal pigment epithelium, ICGA: indocyanine green angiography, GLD: greatest linear dimension. Since the “polypoidal CNV” had been proposed for CNV in PCV or PCV itself and the usage of “POLYPOIDAL CNV” by Yuzawa et al. [13,14] differs from the originally used term “polypoidal CNV”, “POLYPOIDAL CNV” by Yuzawa et al. [13,14] is written in uppercase.

**Table 2:** Characteristics of type 1 PCV and type 2 PCV.

	Type 1 PCV	Type 2 PCV
Miki et al. [18] (Japan)	*N* = 81 (46%)	*N* = 94 (54%)
	Significant associations with *CFH* I62V and *ARMS2* A69S	Significant association with *CFH* I62V but not with *ARMS2* A69S
Yanagisawa et al. [19] (Japan)	*N* = 152 (37%)	*N* = 259 (63%)
	No significant association with *ELN* SNPs	Significant associations with *ELN* SNPs
Honda et al. [20] (Japan)	*N* = 42 (45%)	*N* = 51 (55%)
	Significantly larger GLD (4333 ± 1188 µm)	Significantly smaller GLD (3075 ± 1443 µm)
	No significant VA improvement after PDT	Significant VA improvement after PDT
Tanaka et al. [17] (Japan)	*N* = 10 (31%)	*N* = 22 (69%)
	Choroidal thickness = 177.5 µm	Choroidal thickness = 266.8 µm
	Branching vascular network dimension = 4.3 mm^2^	Branching vascular network dimension = 1.07 mm^2^
	OCTA could clearly depict polypoidal lesions in 17% of type 1 PCV.	OCTA could clearly depict polypoidal lesions in 46% of type 2 PCV.
	The polyps were located immediately beneath the RPE in OCTA.	The polyps were located in outer retina, beneath the RPE, and in choroid in OCTA.
	The branching vascular network was located between the RPE and Bruch’s membrane in OCTA.	The branching vascular network was located between the RPE and Bruch’s membrane or in choroid in OCTA.
Cheng et al. [21] (China)	*N* = 35 (60%)	*N* = 23 (40%)
	Less VA improvement after conbercept	More VA improvement after conbercept
	Less CRT decrease after conbercept	More CRT decrease after conbercept
Nakai et al. [22] (Japan)	*N* = 18 (47%)	*N* = 20 (53%)
	Similar GLD (4591 ± 1622 µm)	Similar GLD (3896 ± 1687 µm)
	Significant VA improvement after ranibizumab	No significant VA improvement after ranibizumab
Sakamoto et al. [23] (Japan)	*N* = 12 (57%)	*N* = 9 (43%)
	Similar VA improvement after aflibercept	Similar VA improvement after aflibercept
	GLD in FA = 3400 ± 1300 µm	GLD in FA = 2400 ± 1100 µm
Yeung et al. [24] (Taiwan)	*N* = 20 (26%)	*N* = 56 (74%)
	Worse baseline VA	Better baseline VA
Kim et al. [25] (South Korea)	*N* = 76 (26%)	*N* = 217 (74%)
	Higher incidence of fibrotic scars after ranibizumab or aflibercept	Lower incidence of fibrotic scars after ranibizumab or aflibercept

PCV: polypoidal choroidal vasculopathy, SNP: single nucleotide polymorphism, GLD: greatest linear dimension, PDT: photodynamic therapy, OCTA: optical coherence tomography angiography, RPE: retinal pigment epithelium, VA: visual acuity, FA: fluorescein angiography.

**Table 3:** Characteristics of larger PCV and smaller PCV.

Larger PCV	Smaller PCV
*N* = 66 (75%)	*N* = 22 (25%)
Worse baseline VA	Better baseline VA
Worse VA change after PDT and/or anti-VEGF treatment	Better VA change after PDT and/or anti-VEGF treatment
GLD in ICGA = 3915 ± 1591 µm	GLD in ICGA = 1901 ± 464 µm

PCV: polypoidal choroidal vasculopathy, VA: visual acuity, PDT: photodynamic therapy, VEGF: vascular endothelium growth factor, GLD: greatest linear dimension, ICGA: indocyanine green angiography.

**Table 4:** Characteristics of eyes with nAMD-related polyps and eyes with idiopathic polyps.

nAMD-Related Polyps	Idiopathic Polyps
*N* = 17 (33%)	*N* = 34 (67%)
Worse baseline VA	Better baseline VA
Drusen prevalence = 100%	Drusen prevalence = 15%
GLD in ICGA = 3292 ± 1542 µm	GLD in ICGA = 1648 ± 913 µm
Thinner choroidal thickness (177 ± 63 µm)	Thicker choroidal thickness (278 ± 100 µm)

AMD: age-related macular degeneration, VA: visual acuity, GLD: greatest linear dimension, ICGA: indocyanine green angiography.

**Table 5:** Association of CVH with treatment outcome of AMD.

	PCV w/CVH	PCV w/o CVH	tnAMD w/CVH	tnAMD w/o CVH	nAMD w/CVH	nAMD w/o CVH
Maruko et al. [36] (Japan)	*N* = 10 (62%)	*N* = 6 (38%)				
	More retinal thinning after PDT	Less retinal thinning after PDT				
Maruko et al. [37] (Japan)	*N* = 15 (56%)	*N* = 12 (44%)				
	Less recurrence after ranibizumab and PDT	More recurrence after ranibizumab and PDT				
Koizumi et al. [35] (Japan)	*N* = 31 (35%)	*N* = 58 (655%)				
	Less dry macular after ranibizumab	Drier macular after ranibizumab				
Cho et al. [38] (South Korea)	*N* = 41 (40%)	*N* = 62 (60%)				
	No significant VA improvement after ranibizumab or bevacizumab	Significant VA improvement after ranibizumab or bevacizumab				
Sonoda et al. [39] (Japan)	*N* = 21 (50%)	*N* = 21 (50%)				
	Less central foveal thickness reduction after ranibizumab	More central foveal thickness reduction after ranibizumab				
Hata et al. [40] (Japan)					*N* = 46 (35%)	*N* = 87 (655%)
					Less dry macula after ranibizumab	Drier macula after ranibizumab
					*N* = 27 (33%)	*N* = 56 (67%)
					Similar dry macula rate after aflibercept	Similar dry macula rate after aflibercept
Nomura et al. [41] (Japan)					*N* = 9	*N* = 16
					Less VA improvement	More VA improvement
Kim et al. [42] (South Korea)	*N* = 35 (53%)	*N* = 31 (47%)				
	Similar dry macula rate after ranibizumab or bevacizumab	Similar dry macula rate after ranibizumab or bevacizumab				
Yanagi et al. [43] (Singapore)	*N* = 31 (43%)	*N* = 41 (57%)				
	More VA improvement after anti-VEGF monotherapy or combination therapy with PDT	Less VA improvement after anti-VEGF monotherapy or combination therapy with PDT				
Ogasawara et al. [44] (Japan)	*N* = 21 (33%)	*N* = 43 (67%)	*N* = 12 (27%)	*N* = 33 (73%)		
	More VA improvement after aflibercept	Less VA improvement after aflibercept	Slightly less VA improvement after aflibercept	Slightly more VA improvement after aflibercept		
Baek et al. [45] (South Korea)	*N* = 33 (38%)	*N* = 53 (62%)				
	Drier macula after bevacizumab and PDT	Less dry macula after bevacizumab and PDT				

CVH: choroidal vascular hyperpermeability, AMD: age-related macular degeneration, PCV: polypoidal choroidal vasculopathy, PDT: photodynamic therapy, VA: visual acuity, VEGF: vascular endothelium growth factor.

**Table 6:** Diagnostic criteria for pachychoroids.

Pachychoroid Is Diagnosed If All of the Following Criteria Are Met
1. Reduced fundus tessellation on color fundus photographs
2. Pachyvessels on OCT and ICGA images (Pachyvessels, pathologically dilated outer choroidal vessels, were defined by the presence of dilated outer choroidal vessels causing attenuation and thinning of the choriocapillaris and Sattler vessels overlying the pachyvessels on OCT images. Moreover, on ICGA images, the dilated choroidal vessels were defined as extending along the entire course of the vessel back to at least one of the ampullae of the vortex vein.)
3. No soft drusen (total area of >125-μm circle) except for pachydrusen (Pachydrusen are yellow-white deposits with irregular round or ovoid shapes, whose outer contour is well-defined and sometimes undercut and/or eroded. Pachydrusen show a scattered distribution over the posterior pole and occur in isolation or in groups of only a few drusen.)
4. CSC characteristics: RPE abnormality independent of MNV lesion, CVH, or a history of CSC

OCT: optical coherence tomography, ICGA: indocyanine green angiography, CSC: central serous chorioretinopathy, RPE: retinal pigment epithelium, MNV: macular neovascularization, CVH: choroidal vascular hyperpermeability.

**Table 7:** Prevalence of polypoidal lesions in PNV and nAMD.

	Study Area	PNV		nAMD		*p* Value †
		*N* *	Polypoidal Lesion Prevalence	*N* *	Polypoidal Lesion Prevalence	
Fung et al. [46]	USA	22	36%			
Pang et al. [48]	USA	3	100%			
Dansingani et al. [56]	USA	22	18%			
Dansingani et al. [57]	USA	18	17%			
Miyake et al. [50]	Japan	39 (20%)	56%	161 (80%)	43%	0.11
Hata et al. [51]	Japan	9 (30%)	44%	21 (70%)	33%	0.69
Matsumoto et al. [52]	Japan	42 (41%)	67%	60 (59%)	50%	0.09
Terao et al. [58]	Japan	18	22%	18	50%	0.16
Azar et al. [59]	Lebanon, etc.	5	40%			
Azuma et al. [60]	Japan	21 (38%)	52%	34 (62%)	24%	0.03
Tagawa et al. [61]	Japan	99	56%			
Eldandi et al. [62]	Japan	27 (30%)	48%	63 (70%)	51%	0.82
Miki et al. [63]	Japan	42	48%			

*N*: number of patients for the study by Fung et al. [46] and number of eyes for the other studies. PNV: polypoidal neovasculopathy, nAMD: neovascular age-related macular degeneration. * When the study design allowed a comparison of the rate of PNV and nAMD, the rates are noted in parenthesis. † *p* value comparing the polypoidal lesion prevalence between PNV and nAMD.

**Table 8:** Treatment response of PNV with or without polypoidal lesions and nAMD with or without polypoidal lesions.

	PNV w/or w/o Polypoidal Lesions	nAMD w/ or w/o Polypoidal Lesions
Miyake et al. [50]	*N* = 28	*N* = 86
	Similar dry macula rate (ranibizumab)	Similar dry macula rate (ranibizumab)
	Longer retreatment-free periods after loading treatment	Shorter retreatment-free periods after loading treatment
Matsumoto et al. [52]	*N* = 42	*N* = 60
	Better baseline VA	Worse baseline VA
	Similar VA improvement (aflibercept)	Similar VA improvement (aflibercept)
	Similar retinal thickness change (aflibercept)	Similar retinal thickness change (aflibercept)
	Similar number of injections (aflibercept)	Similar number of injections (aflibercept)
	Fewer number of injections in PNV with polypoidal lesions than PNV without polypoidal lesion	
Terao et al. [58]	*N* = 18	*N* = 18
	Similar dry macula rate (aflibercept)	Similar dry macula rate (aflibercept)
Azuma et al. [60]	*N* = 21	*N* = 34
	Similar CNV area	Similar CNV area
	Similar dry macular rate (ranibizumab or aflibercept)	Similar dry macular rate (ranibizumab or aflibercept)
	Similar VA improvement (ranibizumab or aflibercept)	Similar VA improvement (ranibizumab or aflibercept)
Elfandi et al. [62]	*N* = 27	*N* = 63
	Similar baseline VA	Similar baseline VA
	Similar GLD (3294 ± 1649 µm)	Similar GLD (3885 ± 1739 µm)
	Similar VA improvement (aflibercept)	Similar VA improvement (aflibercept)
Sacconi et al. [84]	*N* = 30	
	PDT was effective	
	*N* = 13	
	Ranibizumab was effective.	
Miki et al. [63]	*N* = 42	
	PDT + ranibizumab/aflibercept was effective.	
	Similar VA, SCT, and CRT between PNV with polypoidal lesions and PNV without polypoidal lesions	
	Fewer additional treatments after the initial treatment for PNV without polypoidal lesions than PNV with polypoidal lesions	

PNV: pachychoroid neovasculopathy, nAMD neovascular age-related macular degeneration, VA: visual acuity, CNV: choroidal neovascularization, SCT: subfoveal choroidal thickness, CRT: central retinal thickness.

**Table 9:** Treatment response of PNV without polypoidal lesions and nAMD without polypoidal lesions.

	PNV w/o Polypoidal Lesions	nAMD w/o Polypoidal Lesions
Cho et al. [72]	*N* = 22	*N* = 183
	Better baseline VA	Worse baseline VA
	Similar VA improvement (ranibizumab or aflibercept)	Similar VA improvement (ranibizumab or aflibercept)
	Similar retinal thickness reduction (ranibizumab or aflibercept)	Similar retinal thickness reduction (ranibizumab or aflibercept)
	Fewer additional treatments	More additional treatments
Yoon et al. [77]	*N* = 41	*N* = 56
	Similar VA change (ranibizumab or aflibercept)	Similar VA change (ranibizumab or aflibercept)
	Fewer injections	More injections
Roy et al. [85]	*N* = 6	
	PDT w/or w/o ranibizumab was effective.	
Jung et al. [73]	*N* = 54	
	Similar VA improvement after ranibizumab and aflibercept	
	Drier macula after aflibercept than ranibizumab	
Schworm et al. [80]	*N* = 21	
	More retinal thickness reduction by additional 3-monthly injection of ranibizumab or aflibercept after three monthly loading injections	
Matsumoto et al. [65]	*N* = 21	
	Half-fluence PDT combined with aflibercept was effective.	
Schworm et al. [81]	*N* = 14	
	Switching from ranibizumab to aflibercept was effective.	
Kitajima et al. [86]	*N* = 11	
	Ranibizumab + PDT was effective.	
Hikichi et al. [66]	*N* = 82	
	Similar VA improvement (PDT vs. ranibizumab/aflibercept)	
Tanaka et al. [64]	*N* = 27	
	Two-thirds dose PDT was effective.	

PNV: pachychoroid neovasculopathy, nAMD neovascular age-related macular degeneration, VA: visual acuity, PDT: photodynamic therapy.

**Table 10:** Treatment response of PCV with pachychoroid and PCV without pachychoroid.

	PCV with Pachychoroid	PCV without Pachychoroid
Hata et al. [87]	*N* = 70 (44%)	*N* = 88 (56%)
	Similar baseline VA	Similar baseline VA
	Similar GLD (4584 ± 1616 µm)	Similar GLD (4139 ± 1867 µm)
	Better final VA after initial PDT followed by PDT or ranibizumab or aflibercept	Worse final VA after initial PDT followed by PDT or ranibizumab or aflibercept
Shimizu et al. [88]	*N* = 48 (42%)	*N* = 67 (58%)
	Similar baseline VA	Similar baseline VA
	Similar final VA after ranibizumab or aflibercept	Similar final VA after ranibizumab or aflibercept

PCV: polypoidal choroidal vasculopathy, VA: visual acuity, GLD: greatest linear dimension, PDT: photodynamic therapy.

## Data Availability

Not applicable.
